# A Nationwide Study on How COVID-19 Changed APS Policies and Practices

**DOI:** 10.1093/geroni/igab046.281

**Published:** 2021-12-17

**Authors:** Pamela Teaster

**Affiliations:** Virginia Tech, BLACKSBURG, West Virginia, United States

## Abstract

The purpose of this inquiry by the Virginia Tech Center for Gerontology and WRMA, Inc., was to explore changes being implemented by APS programs across the country in response to the COVID-19 pandemic. With input from the Administration for Community Living, the research team used a three-step process (e.g., telephone interviews with state-level APS administrators, a national online survey, and in-depth interviews with local and APS) to capture information on changes caused by efforts to mitigate the spread of COVID-19. This presentation concerns changes in APS policy and practice that the pandemic caused, including modifications in-person visits and adjustments to timeline requirements. Discussion of alterations in policy and practices during the first five months of the pandemic can eludicate APS and other services and planning for older adults in future emergency situations.

